# 

**Published:** 1857-06

**Authors:** 


### DENTAL REGISTER

### OF THE WEST.

EDITED BY

J. TAFT AND GEO. WATT.

June, 1857.

Published Quarterly at $3 Per Annum.

CINCINNATI:

WRIGHTSON & CO., PRINTERS.

